# Mechanical Performance of Steel Slag Concrete under Biaxial Compression

**DOI:** 10.3390/ma13153268

**Published:** 2020-07-23

**Authors:** Xiaoyan Wen, Jingkai Zhou, Beiquan Chen, Zhiheng Deng, Bing Liu

**Affiliations:** 1 College of Civil Engineering and Architecture, Guangxi University, Nanning 530004, China; gxu_wxy@163.com (X.W.); gxu_zjk@163.com (J.Z.); 2 Key Laboratory of Disaster Prevention and Structural Safety of Ministry of Education, Guangxi University, Nanning 530004, China; 3 Guangzhou Design Institute, Guangzhou 510620, China; gzdi_cbq@163.com

**Keywords:** steel slag concrete, biaxial compression, replacement ratio, stress ratio, strength failure criterion

## Abstract

The mechanical performance of steel slag concrete (SSC) under biaxial compression is investigated by a servo-controlled static-dynamic true triaxial machine (TAWZ-5000/3000). Three replacement ratios of steel slags and four kinds of stress ratio (0.25:1, 0.5:1, 0.75:1, and 1:1) are examined in this study. According to the test results, the influences of replacement ratio and stress ratio on the strength, deformation properties, stress–strain curves, and failure mode of SSC are analyzed. The results show that the failure mode of SSC under biaxial compression is plate-splitting crack. Both the strength and deformation of SSC are larger than the corresponding values of the uniaxial compression. Under the same stress ratio, the value of principal stress $\sigma_{3f}$ increases first and then decreases with the increase in the replacement ratio. Under the same replacement ratio, $\sigma_{3f}$ increases first and then decreases as the stress ratio increases, and the maximum of $\sigma_{3f}$ is obtained at the stress ratio of α= 0.5:1. Based on the analysis and test data, the strength failure criterion of SSC under biaxial compression stresses is proposed.

## 1. Introduction

Steel slags, which are by-products of steel-making, are annually produced in a huge quantity, and landfilling steel slag will not only occupy land resources but also pollute the environment [1,2]. Although the recycling rate of steel slag covers 65% in Europe [2,3], it is still very low in developing countries, such as China and Vietnam [1,4,5]. The recycling of steel slag still is a problem. Previous results show that the steel slag has some hydraulic activity, and the crushing resistance and freeze-thaw resistance of steel slags are similar to or even better than those of ordinary aggregates. Thus, the steel slag is considered a desired substitute for natural aggregate [6,7,8,9]. At the same time, large usage of crushed stones and river sands will throw delicate ecosystems off balance. The use of steel slag instead of crushed stone not only alleviates the shortage of concrete resources, but also solves the problems of steel slag reuse and environmental damage. Currently, steel slags are mainly used in road construction, cement and concrete, and asphaltic concrete [10]. At the same time, steel slag concrete (SSC) can be used in construction areas with special requirements due to the characteristics of steel slag [11]. It has great significance in the application of steel slag in concrete. 

The mechanical properties of SSC under uniaxial compression have been studied by many scholars. The research shows that the steel slag has a rough surface, which promotes better steel slags and cement hydration products integration, therefore the microhardness of the interface transition zone (ITZ) in SSC is higher than that of in ordinary concrete [12,13,14,15,16]. So, the mechanical properties of SSC are better than those of ordinary concrete under uniaxial compression [10,16,17,18,19]. The SSC has a certain engineering application value. However, the steel slag replacement ratio and the size of the steel slag should be limited in a certain scope [20,21]. Because the MgO and CaO in steel slags will expand during the process of hydration, resulting in internal damage to the concrete, the higher the steel slag replacement ratio, the greater the internal damage [22,23]. Currently, the studies of SSC under multiaxial loading and the effects of steel slag replacement ratio on the performance of SSC under multiaxial loading are extremely rare.

Numerous studies show that the mechanical properties of concrete under multiaxial stresses are significantly different from those of under uniaxial stress. The failure mode of concrete under uniaxial compression is columnar failure, but it turns to plate-splitting crack under biaxial compression. The strength and peak strain of concrete under biaxial compression is higher than that of under uniaxial compression [24,25,26]. However, in practical engineering, especially in bridges, dams and nuclear power plant containment structures, concrete members are subjected to multiaxial loading. The theory of structure design and examination based on uniaxial force will lead to material waste or insufficient safety. The study on the mechanical properties of concrete under multiaxial stresses is particularly important. Numerous studies on those show that the mechanical properties of different types of concrete under multiaxial stresses are significantly different, which are related to the concrete category and composition of concrete [25,26,27,28,29,30,31]. In addition, the material properties of steel slag are different from those of nature aggregate, so it is necessary to investigate the mechanical properties of SSC under multiaxial stresses.

In this paper, the mechanical properties of SSC under biaxial compression stresses are studied. The effects of stress ratio and replacement ratio of steel slag on the mechanical properties of SSC under biaxial compression stresses are analyzed and discussed. In addition, based on the experimental data, a failure criterion model for SSC under biaxial compression is proposed. The results of the present work not only can be helpful to better understand the mechanical characteristics of SSC, but also to provide a theoretical basis for its application in practical engineering, as it can save materials and take into account the safety of structures.

## 2. Materials and Methods

### 2.1. Materials

**Figure 1:**
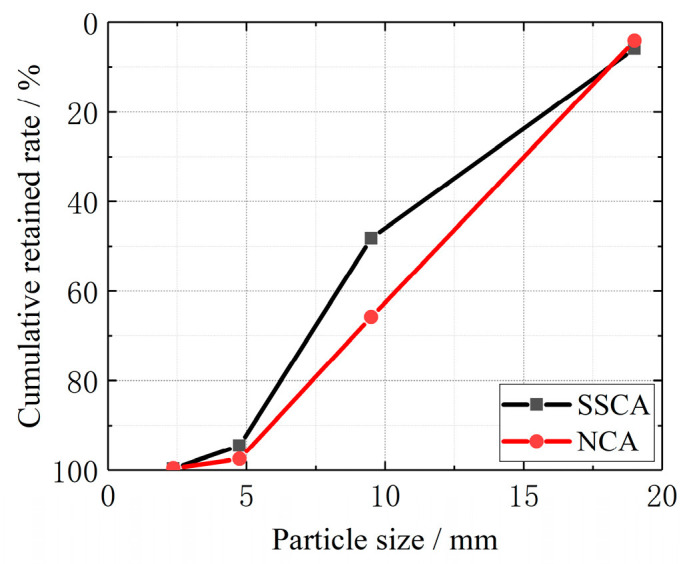
The graining curves for steel slags used as coarse aggregates (SSCA) and natural coarse aggregates (NCA).

The steel slags used in this study were by-products of industrial production from a factory in Liuzhou, China. After a series of treatments, such as crushing and screening, the steel slags were used as coarse aggregates (SSCA). The natural coarse aggregates (NCA) used in this study were crushed stone. The physical properties of both types of coarse aggregate were measured according to GB/14685 [32] and shown in Table 1. The graining curves for SSCA and NCA are shown in Figure 1. River sand was used as fine aggregates. Additionally, P.O. 42.5 Ordinary Portland cement and tap water were also used.

### 2.2. Mix Proportions and Samples

The mix proportions of SSC were designed following the code JGJ55 [33] and shown in Table 2. Three SSCA replacement ratios, 30%, 70%, and 100% by mass, were considered in the mix proportion design.

In this study, four stress ratios (0.25:1, 0.50:1, 0.75:1, and 1:1) were designed for each mix proportion. Meanwhile the corresponding uniaxial compression test was designed as the benchmark. Thus, a total of 45 100×100×100 mm cube specimens were prepared. The materials were mixed in a concrete mixer in order, and then the fresh concrete was cast in the plastic molds and compacted on a vibration table. Specimens were removed from the molds after 24 h and moved to the standard curing room, where the temperature was 20 ± 2 °C and the relative humidity was above 95%, and were cured for 28 days.

### 2.3. Apparatus and Testing Method

**Figure 2:**
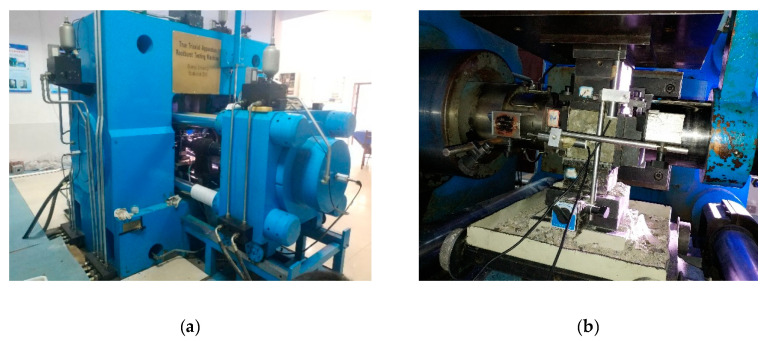
The experimental setup. (**a**) The exterior of the setup; (**b**) the interior of the setup.

**Figure 3:**
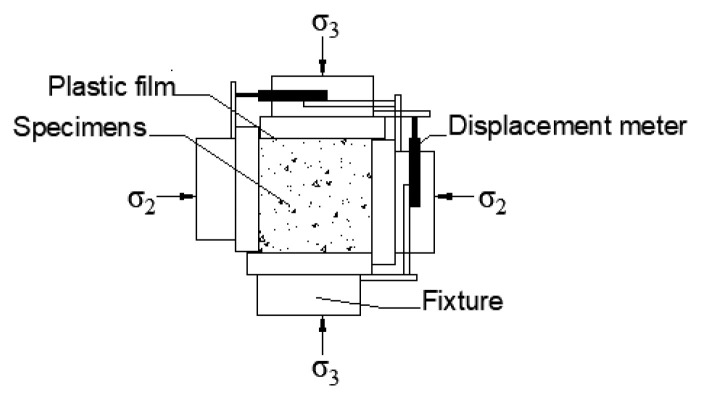
The biaxial loading pattern.

The biaxial compression test was carried out on the servo-controlled static-dynamic true triaxial machine (TAWZ-5000/3000), which is at State Key Laboratory of Structural Engineering in Guangxi University and manufactured by Changchun City Chaoyang Testing Instrument Co. LTD in Changchun, China. The machine consists of six independent actuators in three directions, and possesses the loading capacity up to 3000, 3000, and 5000 kN in the X, Y, and Z direction, respectively. Before the test, the surfaces of specimens were polished to eliminate the effect of uneven force. Two layers of polyethylene films and glycerin were used to eliminate the influence of friction between the specimen and machine [34]. The test setup and loading schematic are shown in Figure 2 and Figure 3, respectively. The displacement sensors were installed along the loading direction to obtain the displacement, and the corresponding data were collected by the servo-controlled system with a preload of 5 kN in each loading direction to eliminate the gap between the fixtures and the specimen before loading. The load in the principal stress direction $\sigma_{3}$ was applied by a force-controlled mode with a rate of 3 kN/s.

## 3. Results and Discussion

### 3.1. Filure Modes

**Figure 4:**
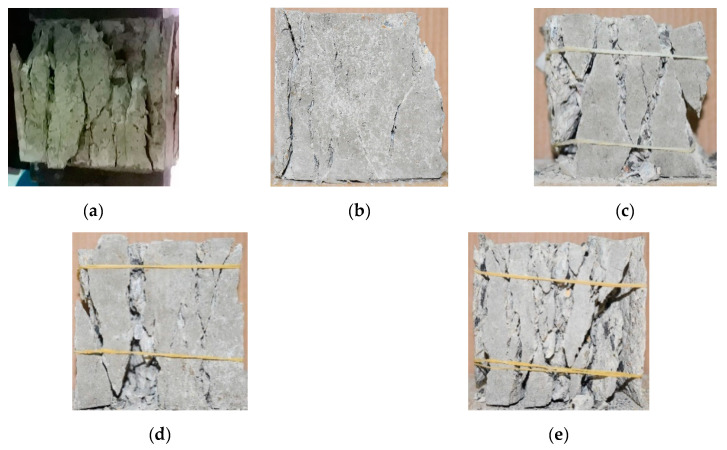
Failure forms of steel slag concrete (SSC). (**a**) 0.00:1; (**b**) 0.25:1; (**c**) 0.50:1; (**d**) 0.75:1; (**e**) 1:1.

The failure modes of SSC specimens under uniaxial and biaxial compression are shown in Figure 4. Under uniaxial compression, the tensile strain was observed along the normal direction of the free surface due to the Poisson’s effect, then cracks parallel to the loading direction appeared on the free surface. Until the crack penetrated, the specimens were divided into pillars of different sizes. The failure mode of SSC under uniaxial compression is typically columnar failure. Under biaxial compression, tensile strain occurs in the normal direction of the free surface due to the constraints of $\sigma_{2}$ and $\sigma_{3}$, and then cracks appear when the deformation reaches its limit. Small angles (20–30°) between macroscopic cracks and the spindle direction were obtained at the stress ratio of α=0.25 ($\alpha =\sigma_{2}$:$\;\sigma_{3}$) due to the influence of coarse aggregates, and the higher the α, the smaller the angle. Cracks were almost perpendicular to the normal direction of the free surface at α=1. The failure mode of SSC is the same as that of ordinary concrete: plate-splitting crack. Note that the SSCA replacement ratio and stress ratio have a slight influence on the failure mode of SSC. However, the number of cracks increased as the SSCA replacement ratio increased, because the brittleness of SSC increases with the SSCA replacement ratio. It is noteworthy that the destruction of ITZ in SSC is different from that of ordinary concrete. When the SSC specimen is damaged, all SSCAs on the failure surface will be broken, and the crushed stone remains intact, but in the ordinary concrete, it is destroyed along the ITZ [5,24]. The rough and porous surfaces of the SSCAs can promote better steel slag and cement hydration products integration. Additionally, the actual water-cement ratio of the ITZ in SSC is lower than that design. Thus, the bonding performance of ITZ in SSC is excellent.

### 3.2. Strength

The test results of SSC under biaxial compression are shown in Table 3. The data in the table are the average value of the three specimens. With reference to references [25,26,27,28,29,30,31,35], the selection of the loading surface was random. The results show that the coefficient of variation of each set of experimental data was not greater than 10%, which indicated that the performance of the SSC was basically the same in every direction. The experimental data in the test are representative.$\;f_{m}$ and $\varepsilon_{m}$ denote the compressive strength and peak strain of the specimens under uniaxial compression,$\;\sigma_{2f}$ and $\sigma_{3f}$ indicate the compressive strength in direction $\sigma_{2}$ and $\sigma_{3}$ of the specimens under biaxial compression, and $\varepsilon_{2p}$ and $\varepsilon_{3p}\;$ represent the peak strain in direction $\sigma_{2}$ and $\sigma_{3}$ of the specimens under biaxial compression. This study stipulates that the tension is positive and the compression is negative.

**Figure 5:**
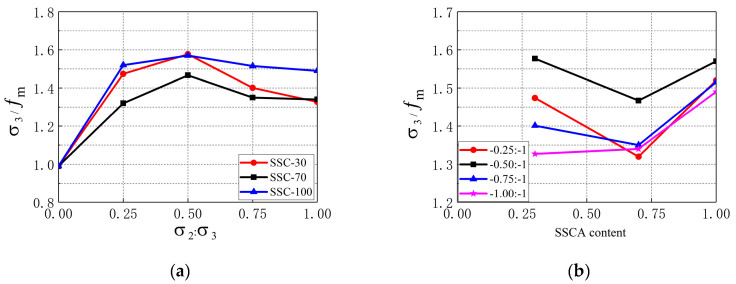
Influence of stress ratio and SSCA replacement ratio on $\sigma_{3f}/f_{m}$. (**a**) $\sigma_{3f}/f_{m}$ with different stress ratios; (**b**) $\sigma_{3f}/f_{m}$ with different SSCA replacement ratios.

Comparing the data in Table 3 with the reference [26], it is found that the strength of SSC under biaxial compression was higher than that of ordinary concrete. Under biaxial compression, the strength of SSC changed with the change of SSCA replacement ratio and stress ratio. It can be seen from Table 3 that the biaxial strength of SSC increased first and then decreased with the increase in SSCA replacement ratio. The maximum strength was obtained when the SSCA replacement ratio was 70%. Some researchers have confirmed that the bonding performance of SSC is higher than that of ordinary concrete due to the rough and porous characteristics of SSCA. However, the free CaO and MgO in SSCA will dilate during the process of hydration, causing uneven expansion stress inside the concrete, which in turn leads to the formation of micro-cracks and the decrease in strength. Figure 5a,b show the influences of stress ratio and SSCA replacement ratio on $\sigma_{3f}/f_{m}$ (relative value of biaxial strength to uniaxial strength), respectively. It can be seen that the value of $\sigma_{3f}/f_{m}$ was between 1.32 and 1.58, which indicated that the biaxial compressive strength of SSC was higher than the uniaxial compressive strength. The reason for this is that the lateral restraint can increase the mechanical interaction force between aggregates, which helps to improve the compressive strength of concrete. From Figure 5a we can learn that the value of $\sigma_{3f}/f_{m}$ increased first and then decreased with the increase in stress ratio regardless of the SSCA replacement ratio. The change trend of SSC strength with stress ratio is the same as that of ordinary concrete [26]. The maximum of $\sigma_{3f}/f_{m}$ was 1.58 and was obtained at α=0.5. As shown in Figure 5b, except for the stress ratio of α=1, the value of $\sigma_{3f}/f_{m}$ decreased with the increase in SSCA replacement ratio when the SSCA replacement ratio was smaller than 70% but increased when the SSCA replacement ratio was changed from 70% to 100%. $\sigma_{3f}/f_{m}$ achieved the maximum when the SSCA replacement ratio was 100%.

### 3.3. Deformation

**Figure 6:**
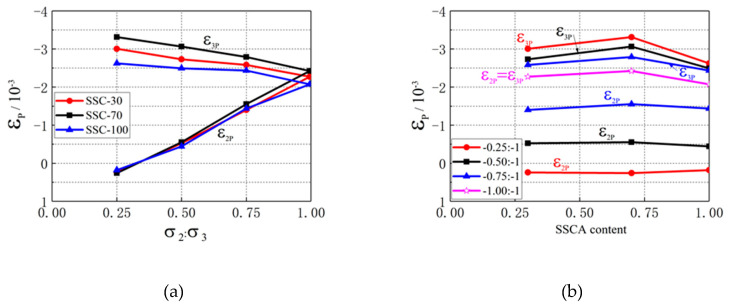
Influence of stress ratio and SSCA replacement ratio on $\varepsilon_{3p}$ and $\varepsilon_{2p}$. (**a**) $\varepsilon_{3p}$ and $\varepsilon_{2p}$ with different stress ratios; (**b**) $\varepsilon_{3p}$ and $\varepsilon_{2p}$ with different SSCA replacement ratios.

Table 3 shows that the peak strain $\varepsilon_{3p}$ was higher than the peak strain $\varepsilon_{m}$. Both the SSCA replacement ratio and stress ratio had great influences on peak strain. The effect of stress ratio on the peak strain of SSC under biaxial compression is shown in Figure 6a. The results revealed that the peak strain $\varepsilon_{3p}$ under all stress ratios was compressive strain. Under the same SSCA replacement ratio, $\varepsilon_{3p}$ decreased nearly linearly when the stress ratio increased. The general trend of $\varepsilon_{3p}$ with stress ratio was $\alpha_{0.25}>\alpha_{0.50}>\alpha_{0.75}>\alpha_{1.00}$. The lateral deformation of concrete was confined due to the action of lateral restraint, and the greater the lateral stress, the more obvious the restraint. The peak strain $\varepsilon_{2p}$ was tensile strain at a stress ratio of α=0.25 due to Poisson’s effect. Then, it was compressive strain when the stress ratio was between 0.25 and 0.50, because the increase in $\sigma_{2}$ enhanced the lateral constraint. $\varepsilon_{2p}$ increased nearly linearly while the stress ratio increased, which is the same as ordinary concrete [26]. The maximum of $\varepsilon_{2p}$, which is $\varepsilon_{2p}=\varepsilon_{3p}$, was obtained at a stress ratio of α=1. Figure 6b shows the influences of SSCA replacement ratio on the peak strain of SSC, it can be seen that $\varepsilon_{3p}$ and $\varepsilon_{2p}$ increased first and then decreased with the increase in SSCA replacement ratio. Except the $\varepsilon_{2p}$ at a stress ratio of α=0.75, the general trends of $\varepsilon_{3p}$ and $\varepsilon_{2p}$ with SSCA replacement ratio were SSC-70 > SSC-30 > SSC-100. The maximums of $\varepsilon_{3p}$ and $\varepsilon_{2p}$ were obtained when the replacement ratio was 70%.

### 3.4. Stress–Strain Relationship

**Figure 7:**
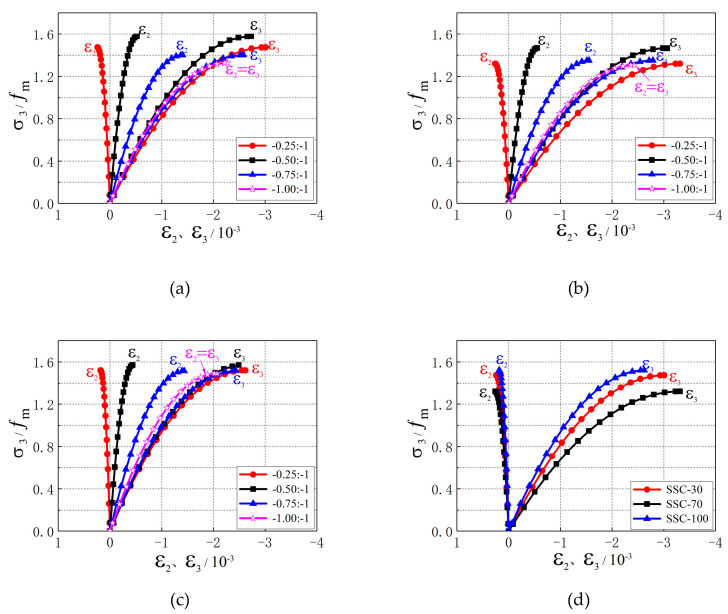

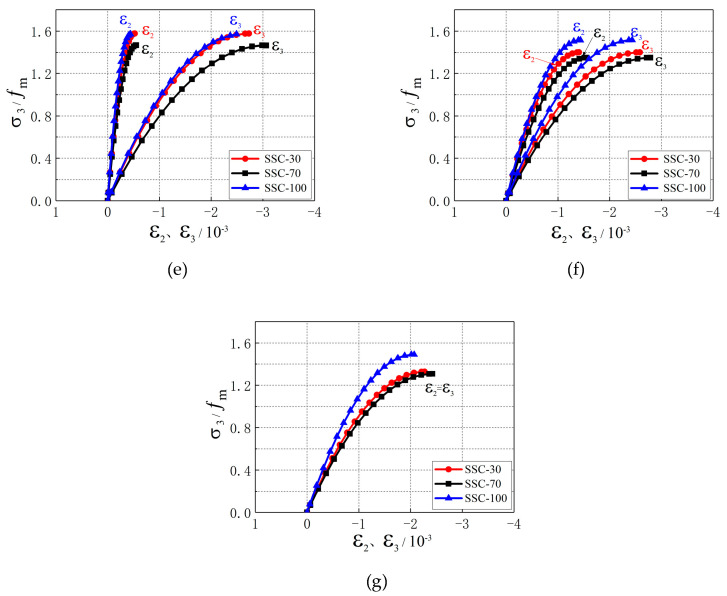
Stress–strain curves of SSC under biaxial compressive stress. (**a**) SSC-30; (**b**) SSC-70; (**c**) SSC-100; (**d**) $\sigma_{2}$:$\;\sigma_{3}\;$= 0.25; (**e**) $\sigma_{2}$:$\;\sigma_{3}$ = 0.50; (**f**) $\sigma_{2}$:$\;\sigma_{3\;}$= 0.75; (**g**) $\sigma_{2}$:$\;\sigma_{3}$ = 1.

Figure 7 shows the stress–strain curve of SSC under biaxial compression. Figure 7a–c show the influences of stress ratio on the stress–strain curve, and Figure 7d–g show the effects of replacement ratio on the stress–strain curve.

As shown in Figure 7, there was an approximately linear relationship between the stress $\sigma_{3}$ and strain $\varepsilon_{3}$ in the initial loading stage ($\sigma_{3\;}$<$\;0.35f_{m}$). With the increase in load, the growth of strain became faster, the relationship between stress $\sigma_{3}$ and strain $\varepsilon_{3}$ changed from linear to nonlinear. When the value of $\sigma_{3}$ reached its peak, the strain $\varepsilon_{3}$ sharply increased. As shown in Figure 7a–c, the slope of the stress–strain curve of $\sigma_{3}$-$\varepsilon_{3}$ increased with stress ratio in the elastic stage, which indicated that the initial elastic modulus of SSC increased with stress ratio. There was an approximately linear relationship between the stress $\sigma_{2}$ and strain $\varepsilon_{2}$ at the stress ratio α=0.25, α=0.50, and α=0.75. The curve of $\sigma_{2}$-$\varepsilon_{2}$ was the same as that of $\sigma_{3}$-$\varepsilon_{3}$ at a stress ratio of α=1. Figure 7d–g show that the SSCA replacement ratio did not change the sign of $\varepsilon_{2p}$. The value of $\varepsilon_{2p}$ increased first and then decreased with the increase in SSCA replacement ratio under the same stress ratio. The initial slope of the stress–strain curve of $\sigma_{3}$-$\varepsilon_{3}$ decreased first and then increased with the increase in SSCA replacement ratio, which indicated that the initial elastic modulus of SSC decreases first and then increases with the increase in SSCA replacement ratio.

### 3.5. Failure Criterion

The common failure criterion of concrete under biaxial compression is put forward by Kupfer [36], which can be expressed as

(1) $${\left(\frac{\sigma_{2f}^{}}{f_{m}}+\frac{\sigma_{3f}^{}}{f_{m}}\right)}^{2}-\frac{\sigma_{3f}^{}}{f_{m}}-3.65\frac{\sigma_{2f}^{}}{f_{m}}=0,$$

where $\sigma_{2f}$ and $\sigma_{3f}$ are the peak stress in the directions $\sigma_{2}$ and $\sigma_{3}$ under biaxial compression, and $f_{m}$ was the average value of uniaxial compressive strength.

**Figure 8:**
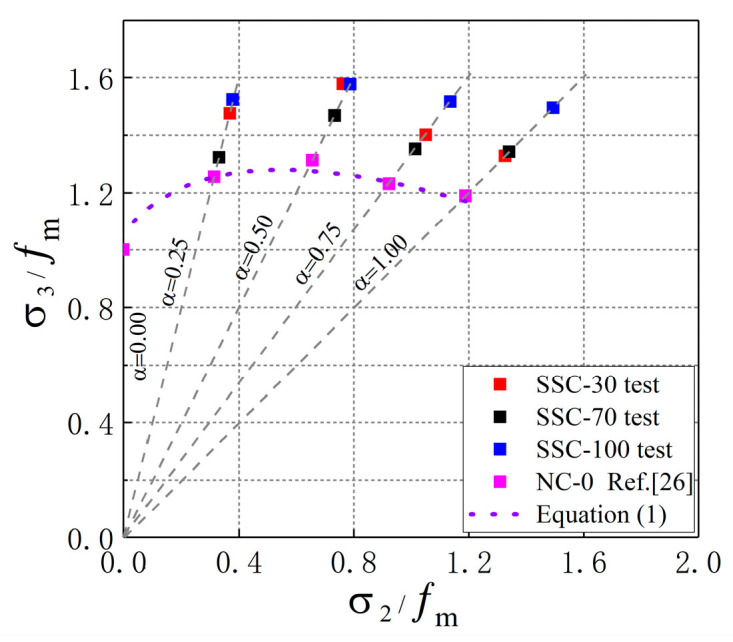
Comparison between Equation (1) and experimental results.

Results of Equation (1) were compared with the test data in this study and the test result of ordinary concrete from other studies [26], as shown in Figure 8. It is shown that the test results in this study were higher than the prediction of Equation (1), which indicates that the prediction of Kupfer’s biaxial failure criterion is too conservative. Meanwhile, this failure criterion was discontinuous at the stress ratio of α=1 [25,37]. Therefore, a failure criterion model suitable for SSC under biaxial compression needs to be established, and the curve of failure criterion must meet the conditions of continuous at the stress ratio of α=1.

The strength envelope of SSC under biaxial compression is similar to the graph of the quadratic curve, thus, the quadratic curve is considered as the expressions of the strength envelope of SSC, which can be expressed as

(2) $$A{\left(\frac{\sigma_{2f}^{}}{f_{m}}\right)}^{2}+B{\left(\frac{\sigma_{3f}^{}}{f_{m}}\right)}^{2}+C\frac{\sigma_{2f}^{}}{f_{m}{}_{}}\cdot \frac{\sigma_{3f}^{}}{f_{m}}+D\frac{\sigma_{2f}^{}}{f_{m}}+E\frac{\sigma_{3f}^{}}{f_{m}}+F=0,$$

where symbols *A*, *B*, *C*, *D*, *E*, and F stand for the parameters. The strength envelope of concrete under biaxial compression is symmetric on the line y=x, so the parameter A=B and D=E in Equation (2). At the same time, *F* can be represented by *A* and *D* because the curve must pass the point (0,1), so Equation (2) can be simplified into Equation (3).

(3) $$A\left[{\left(\frac{\sigma_{2f}^{}}{f_{m}}\right)}^{2}+{\left(\frac{\sigma_{3f}^{}}{f_{m}}\right)}^{2}\right]+C\frac{\sigma_{2f}^{}}{f_{m}{}_{}}\cdot \frac{\sigma_{3f}^{}}{f_{m}}+D(\frac{\sigma_{2f}^{}}{f_{m}}+\frac{\sigma_{3f}^{}}{f_{m}})-(A+D)=0,$$

The parameter *A*, *C*, *D* and the coefficient of determination R^2^ were obtained by analyzing the test data in this study with Equation (3), as shown in Table 4.

**Figure 9:**
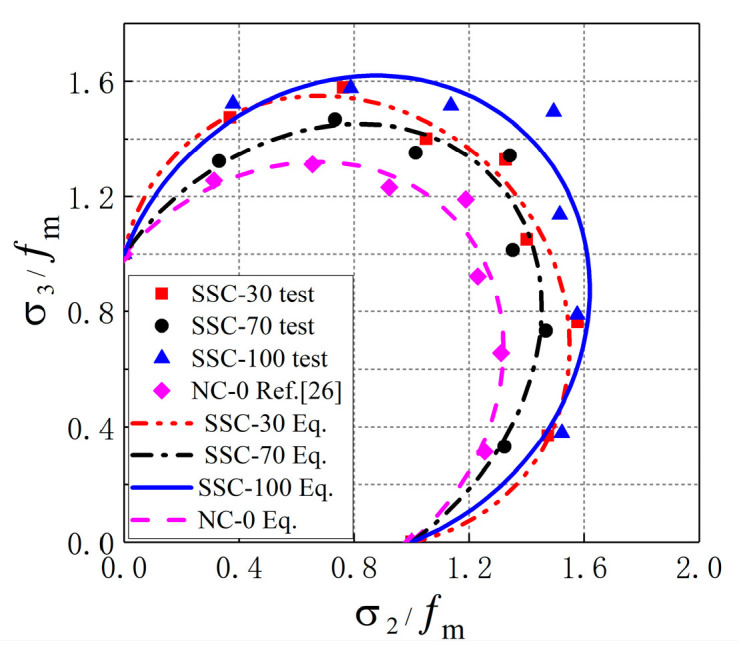
Biaxial C–C failure criterion model of SSC.

According to the data in Table 4, the strength envelope of SSC under biaxial compression was obtained, as shown in Figure 9. It can be seen that the calculated values of the strength failure criterion model Equation (3) were close to the experimental values, which indicates that the failure criterion model proposed in this study is suitable for SSC.

## 4. Conclusions

1. The failure mode under biaxial compression is plate-splitting crack. The angle between the direction of cracks and the principal axis decreases as the stress ratio increases, and the number of cracks increases with SSCA replacement ratio.
2. The biaxial compressive strength of SSC is higher than uniaxial compressive strength. The value of $\sigma_{3f}/f_{m}$ first decreases and then increases with the increase in SSCA replacement ratio except the stress ratio of α=1, and the maximum is obtained at 100% SSCA. The value of $\sigma_{3f}/f_{m}$ first increases and then decreases with the increase in the stress ratio and the maximum is obtained at stress ratio of α=0.5.
3. The peak strain of SSC under biaxial compression is higher than under uniaxial compression. The value of $\varepsilon_{3p}$ decreases nearly linearly with the increase in stress ratio, and the value of $\varepsilon_{2p}$ increases nearly linearly with the increase in stress ratio. The values of $\varepsilon_{3p}$ and $\varepsilon_{2p}$ increase first and then decrease with the increase in SSCA replacement ratio except the $\varepsilon_{2p}$ at a stress ratio of α=0.75.
4. The stress–strain curve of SSC under biaxial compression relates to the stress ratio and SSCA replacement ratio. The initial slope of the curve increases with the increase in stress ratio, while it decreases first and then increases with the increase in SSCA replacement ratio.
5. Based on the analysis of the test data, the strength failure criterion model for SSC is proposed, and the proposed model has a good predictive capacity for SSC under biaxial compression.

## Figures and Tables

**Table 1:** Physical properties of coarse aggregate.

Classification	Gradation (mm)	Bulk Density (kg/m^3^)	Apparent Density (kg/m^3^)	Crush Index (%)	Water Absorption (%)
SSCA	5–20	2280.00	3230.00	6.10	1.36
NCA	5–20	1480.00	2760.00	10.40	0.65

**Table 2:** Mix proportion.

No.	Replacement Ratio (%)	W/C ^1^	Water (kg)	Cement (kg)	Natural Fine Aggregate (kg)	SSCA (kg)	NCA (kg)
SSC-30	30	0.56	200	357	777	350	816
SSC-70	70	0.56	200	357	777	816	350
SSC-100	100	0.56	200	357	777	1166	0

Note: ^1^ W/C = Water/Cement.

**Table 3:** Test value of SSC under uniaxial and biaxial compression.

No.	$\sigma_{2}$ $\text{:}\;\sigma_{3}$	$\sigma_{2f}$ (MPa)	$f_{m}$$\mathrm{or}\;\sigma_{3f}$ (MPa)	$\;\varepsilon_{2p}$ (10^−3^)	$\varepsilon_{m}$$\mathrm{or}\;\varepsilon_{3p}$ (10^−3^)	$\sigma_{3f}/f_{m}$
SSC-30	0.00:1	-	−38.32	-	−2.11	-
	0.25:1	−14.12	−56.49	0.24	−3.01	1.47
	0.50:1	−29.22	−60.44	−0.52	−2.73	1.58
	0.75:1	−40.26	−53.68	−1.41	−2.58	1.4
	1.00:1	−50.86	−50.86	−2.27	−2.27	1.33
SSC-70	0.00:1	-	−43.86	-	−2.29	-
	0.25:1	−14.50	−58.01	0.26	−3.31	1.32
	0.50:1	−32.18	−64.36	−0.55	−3.06	1.47
	0.75:1	−44.47	−59.29	−1.55	−2.79	1.35
	1.00:1	−58.83	−58.83	−2.42	−2.42	1.34
SSC-100	0.00:1	-	−36.17	-	−1.92	-
	0.25:1	−13.70	−55.06	0.18	−2.62	1.52
	0.50:1	−28.49	−56.99	−0.44	−2.49	1.58
	0.75:1	−41.11	−54.81	−1.44	−2.44	1.52
	1.00:1	−54.04	−54.04	−2.07	−2.07	1.49

**Table 4:** The fitting results of failure criterion model for SSC.

Parameter	Specimens			
	SSC-30	SSC-70	SSC-100	NC-0
*A*	1.503	1.206	0.502	1.003
*C*	0.340	−0.541	−0.115	−0.341
*D*	−2.570	−1.177	−0.693	−0.901
R^2^	0.998	0.995	0.994	0.997

## References

[1] Yi H., Xu G.P., Chen H.G., Wang J.S., Wan Y.F., Chen H. (2012). An overview of utilization of steel slag. Procedia Environ. Sci..

[2] Zhang H.W., Hong X. (2011). An overview for the utilization of wastes from stainless steel industries. Resour. Conserv. Recycl..

[3] Motz H., Geiseler J. (2001). Products of steel slags an opportunity to save natural resources. Waste Manag..

[4] Shi C., Qian J. (2000). High performance cementing materials from industrial slags—A review. Resour. Conserv. Recycl..

[5] Nguyen T.T.H., Phan D.H., Mai H.H., Nguyen D.L. (2020). Investigation on compressive characteristics of steel-slag concrete. Materials.

[6] Faleschini F., Alejandro F.M., Zanini M.A. (2015). High performance concrete with electric arc furnace slag as aggregate: Mechanical and durability properties. Constr. Build. Mater..

[7] Sezer G.I., Gülderen M. (2015). Usage of steel slag in concrete as fine and/or coarse aggregate. Indian J. Eng. Mater. Sci..

[8] Montgonmery D.G., Wang G. (1991). Instant-chilled steel slag aggregate in concrete-strength related properties. Cem. Concr. Res..

[9] Maslehuddin M., Sharif A.M., Shameem M. (2003). Comparison of properties of steel slag and crushed limestone aggregate concrete. Constr. Build. Mater..

[10] Jiang Y., Ling T.C., Shi C.J., Pan S.Y. (2018). Characteristics of steel slags and their use in cement and concrete—A review. Resour. Conserv. Recycl..

[11] Banthia N., Djerdane S., Pigeon M. (1995). Electrical resistivity of carbon and steel micro-fiber reinforced cements. Cem. Concr. Res..

[12] Shang J.L., Xing L.L. (2013). Study on Interfacial Transition Zone of Steel Slag Coarse Aggregate Concrete. J. Build. Mater..

[13] Faleschini F., Brunelli K., Zanini M.A., Dabala M. (2016). Electric Arc Furnace Slag as Coarse Recycled Aggregate for Concrete Production. J. Sustain. Metall..

[14] Alexander S.B., Jeffery R.R. (2018). Interfacial transition zone of cement composites with steel furnace slag aggregates. Cem. Concr. Compos..

[15] San-José J.T., Vegas I., Arribas I., Marcos I. (2014). The performance of steel-making slag concretes in the hardened state. Mater. Des..

[16] Pellegrino C., Gaddo V. (2009). Mechanical and durability characteristics of concrete containing EAF slag as aggregate. Cem. Concr. Compos..

[17] Beshr H., Almusallam A.A., Maslehuddin M. (2003). Effect of coarse aggregate quality on the mechanical properties of high strength concrete. Constr. Build. Mater..

[18] Qasrawi H. (2012). Use of relatively high Fe2O3 steel slag as coarse aggregate in concrete. ACI Mater. J..

[19] Qasrawi H. (2014). The use of steel slag aggregate to enhance the mechanical properties of recycled aggregate concrete and retain the environment. Constr. Build. Mater..

[20] Pang B., Zhou Z., Xu H. (2015). Utilization of carbonated and granulated steel slag aggregate in concrete. Constr. Build. Mater..

[21] Pellegrino C., Cavagnis P., Faleschini F., Brunelli K. (2013). Properties of concretes with Black/Oxidizing Electric Arc Furnace slag aggregate. Cem. Concr. Compos..

[22] Mi G.D., Wang Q., Wang W.L. (2015). Destructive effect of steel slag coarse aggregate on the concrete under autoclaved condition. J. Tsinghua Univ. Sci. Technol..

[23] Wang Q., Wang D., Zhuang S. (2017). The soundness of steel slag with different free CaO and MgO contents. Constr. Build. Mater..

[24] Guo Z.H. (2013). Principles of Reinforced Concrete.

[25] Zhou J.J., Pan J.L., Leung C.K.Y., Li Z.J. (2014). Experimental study on mechanical behavior of high performance concrete under multi-axial compressive stress. Sci. China-Technol. Sci..

[26] Rong C., Shi Q.X., Zhang T., Zhao H.C. (2018). New failure criterion models for concrete under multiaxial stress in compression. Constr. Build. Mater..

[27] Deng Z.H., Wang Y.M., Sheng J., Hu X. (2017). Strength and deformation of recycled aggregate concrete under triaxial compression. Constr. Build. Mater..

[28] Deng Z.H., Sheng J., Wang Y.M. (2019). Strength and constitutive model of recycled concrete under biaxial compression. KSCE J. Civ. Eng..

[29] Liu H.Y., Song Y.P. (2010). Experimental study of lightweight aggregate concrete under multiaxial stresses. J. Zhejiang Univ.-Sci..

[30] Yu Z.P., Huan Q., Li F., Qin Y., Zhang J. (2019). Experimental Study on Mechanical Properties and Failure Criteria of Self-Compacting Concrete under Biaxial Tension-Compression. J. Mater. Civ. Eng..

[31] Yu Z.P., Huan Q., Xie X.H. (2019). Experimental study on mechanical properties of ordinary concrete and lightweight aggregate concrete based on biaxial loading. J. Build. Mater..

[32] (2011). The State Standard of China. Pebble and Crushed Stone for Construction (GB/T14685-2011).

[33] (2011). The Profession Standard of China. Specification for Mix Proportion Design of Ordinary Concrete (JGJ 55-2011).

[34] Wang C.Z., Guo Z.H., Zhang X.Q. (1987). Experimental investigation of biaxial and triaxial compressive concrete strength. ACI Mater. J..

[35] Wang H.L., Song Y.P. (2008). Failure criterion of concrete with different aggregate gradations under biaxial compression. J. Hydraul. Eng..

[36] Kupfer H., Gerstle K.H. (1973). Behavior of concrete under biaxial stresses. ASCE.

[37] Yu Z.P., Shan Y.S., Huan Q., Sun X. (2019). Failure criteria and mechanical properties of recycled concrete with different replacement ratios of coarse aggregates under multiaxial compression. J. Mater. Civ. Eng..

